# Constitutional delay of puberty: presentation and inheritance pattern in 48 familial cases

**DOI:** 10.1186/s12887-016-0580-3

**Published:** 2016-03-12

**Authors:** Sarah Winter, Aldjia Ousidhoum, Kenneth McElreavey, Raja Brauner

**Affiliations:** Université Paris Descartes and Fondation Ophtalmologique Adolphe de Rothschild, Paris, France; Human Developmental Genetics, Institut Pasteur, Paris, France

**Keywords:** CDP constitutional delay of puberty, GnRH, Hypothalamic-pituitary-gonadal axis, Pubertal delay, Unilineal and bilineal inheritance, Autosomal dominant inheritance

## Abstract

**Background:**

The mechanism that initiates the onset of puberty is largely unknown but the age of onset is mainly under genetic control and influenced by environmental factors including nutrition. Familial forms of constitutional delay of puberty (CDP) suggest the involvement of genetic factors. The purpose of this study is to describe the presentation and the mode of inheritance of CDP in a series of familial cases.

**Methods:**

A retrospective, single center study was carried out over 10 years on 48 probands (14 girls and 34 boys) from 48 families seen for CDP with a familial component.

**Results:**

Of the 48 probands, 46 (96 %) had at least one affected 1^st^ degree relatives and 2 (4 %, 2 boys) had only 2^nd^ degree relatives affected.

In girls, 11 families (79 %) exhibited exclusive maternal inheritance, 1 (7 %) paternal inheritance and 2 (14 %) both maternal and paternal inheritance. In boys, 14 families (41 %) exhibited exclusive maternal inheritance, 12 (35 %) paternal inheritance and 8 (24 %) both maternal and paternal inheritance.

In the boys with bilineal inheritance, the ages at onset of puberty (16 ± 1.41 years) and at evaluation (16.05 ± 2.47 years) were higher than in those with unilineal inheritance (15.25 ± 0.35 and 15.1 ± 0.42 years respectively), but the difference was not significant.

**Conclusions:**

In girls exclusive maternal inheritance seems to be the major mode of inheritance whereas for boys the mode of inheritance was almost equally maternal, paternal or bilineal. Clinical phenotype of boys with bilineal inheritance seems to be more severe, but the difference did not reach statistical significance, perhaps because of the small sample size. This greater severity of the phenotype in boys with bilineal inheritance is likely due to inheriting different puberty timing genes from each parent. Future research should be directed at identifying such genes.

## Background

Delayed puberty is diagnosed when there is no testicular enlargement after 14 years in boys or breast development after 13 years in girls. The constitutional delay of puberty (CDP) can be diagnosed only after underlying conditions have been ruled out and when the pubertal delay is followed by spontaneous pubertal development [1]. Boys are affected much more frequently than girls, while the reverse is true in idiopathic central precocious puberty [2].

The age at onset and the progression of puberty are driven by a complex interaction between genetic, metabolic and environmental factors. The age at onset of puberty is often homogeneous within the family and concordant between twins [3]. Kaprio et al. [4] analyzed the age at menarche in monozygotic twins and showed that 74 % of the variance is due to genetic factors and 26 % to environmental factors. The frequency of the familial forms has been reported at 56–80 % in CPD [5] while they represent 41 % in our series of 493 girls with central precocious puberty [6]. Two studies analyzed the inheritance of CDP [7, 8]. The studies [7–10] suggested an autosomal dominant mode, without the influence of obesity.

We evaluated 48 patients seen consecutively by the same senior pediatric endocrinologist for CDP with a familial component. The purpose of this study is to describe the presentation and the mode of inheritance of CDP in a series of familial cases.

## Methods

### Compliance with the ethical standards

The protocol was approved by the Ethical Review Committee Comité de Protection des Personnes Ile de France III (certificate AC 048 joined). All of the clinical investigations were conducted according to the principles expressed in the Declaration of Helsinki. Written informed consent for the evaluations was obtained from the children’s parents and was included in their hospital medical records. No additional activity to routine patient care was performed.

### Patients

This retrospective, single center study was carried out on 48 probands (14 girls and 34 boys) from 48 families seen for CDP with a familial component by a senior pediatric endocrinologist (R Brauner) in a university pediatric hospital from 2001 to 2011 (over 10 years). CDP was diagnosed on the absence of pubertal development after 14 years in boys and 13 years in girls after exclusion of other diseases (see below) and followed by spontaneous pubertal development [1]. The diagnosis of delayed puberty assigned to the individuals in the parental and grandparental generations was based on interviews and recall.

### Methods

First-degree relatives were defined as mother, father, brother(s) and sister(s); second-degree relatives as grandparents, aunt(s) and uncle(s). The familial probands were further subdivided into two groups: 1) unilineal families (only one affected parent and no evidence for delayed puberty in the other parent or his/her first-degree relatives); 2) bilineal families (either both parents affected or an unaffected parent having an affected sibling or parent). Families with lineal maternal are those in which at least one member of the mother’s family is affected, and no father’s family, with similar schema for the lineal paternal involvement.

No patient had suffered from intrauterine growth retardation, a disease or any symptoms.

The onset of puberty is defined as the appearance of breast buds in girls and a testicular volume index of 4 cm^2^ or greater in boys. The dimensions of the testes were measured by a clinical evaluation, the testicular volume index (cm^2^) being length x width [11]. The pubertal stage was rated according to Marshall and Tanner [12, 13]. Body mass index (BMI) (weight in kg/height in m squared) was expressed as the standard deviation score (SDS) for chronological age [14, 15]. Bone age (BA) was assessed in 39 cases (by R Brauner) using the Greulich and Pyle method [16].

The other evaluations were performed to 1) exclude abnormalities of the hypothalamic-pituitary-gonadal axis, and of the uterus in girls, 2) evaluate the pubertal stage, and 3) exclude general diseases responsible for the delayed puberty.

The hypothalamic-pituitary-gonadal axis was evaluated by basal and gonadotropin-releasing hormone (GnRH)-stimulated plasma concentrations of luteinizing hormone (LH) and follicle-stimulating hormone (FSH). The basal plasma LH and FSH concentrations were normal, against primary gonadal failure. The GnRH test, consisting of an intravenous injection of GnRH (100 μg/m^2^) and measurement of LH and FSH peaks at 0, 30, 60 and 90 min, was performed in 4 girls ad 11 boys. The results excluded gonadotropin deficiency. The following values were considered to be pubertal: uterus length ≥ 35 mm [17], LH/FSH peak ratio after GnRH test ≥ 0.66 in girls and ≥ 2 in boys [18], and plasma estradiol concentrations ≥ 15 pg/mL in girls and testosterone ≥ 0.5 ng/mL in boys. Blood samples were collected at 14.05 ± 0.35 (13–16) years in girls and 15.9 ± 2.69 (14–17.8) years in boys, explaining the wide variations in the pubertal stage, increase in LH and FSH after the GnRH test, and plasma testosterone or estradiol concentrations. Plasma estradiol and testosterone concentrations were measured with a radioimmunoassay. Pelvic ultrasonography was performed in 5 girls (cases 7, 11, 12, 13, and 14).

General diseases responsible for delayed puberty and/or short stature were excluded. The plasma concentrations of anti-transglutaminase antibodies and blood creatinine, erythrocyte sedimentation rate, and a sweat test if there was any clinical doubt were normal. A growth hormone (GH) stimulation test was performed in 6 girls and 7 boys with decreased growth rate to exclude GH deficiency. The plasma insulin-like growth factor (IGF) 1 concentration was measured in the majority of the patients (Table 1), as well as basal plasma prolactin. A cerebral magnetic resonance imaging (MRI) was performed in 8 girls and 15 boys with a low GH peak during the second stimulation test and/or a low IGF 1 concentration to evaluate the hypothalamic-pituitary region and to exclude olfactory bulb hypoplasia. In the patients with rapidly increasing weight, we measured the plasma free thyroxin and thyroid stimulating hormone concentrations to exclude hypothyroidism and 24-h urinary cortisol to exclude hypercorticism. If there was any clinical doubt, x-rays of the pelvis and lumbar spine were taken (front and profile) to exclude constitutional bone disease. All of these investigations showed normal results. All of the patients were followed until complete pubertal development. Those with low BMI were encouraged to increase their caloric intake.

**Table 1:** Characteristics at presentation of 48 probands with familial constitutional delay of puberty

	GIRLS (*n* = 14)		BOYS (*n* = 34)		*p*
	*n*	m ± SD	*n*	m ± SD	
Age at onset, years	14	13.65 ± 0.77	29	15.65 ± 1.9	< 0.01
Age at evaluation, years	14	14.05 ± 0.35	34	15.1 ± 0.42	< 0.01
BMI, SDS	14	−1.5 ± 0.7	34	−2 ± 1.41	NS
BA delay, years	12	2 ± 1.13	27	3.05 ± 2.4	NS
Tanner pubic hair	13	3 ± 0	33	2.5 ± 0.7	NS
Age at menarche in mothers, years	13	15.75 ± 0.35	30	14.25 ± 1.8	0.01
IGF 1, ng/mL	12	190.5 ± 77	28	233 ± 83	NS
LH peak, IU/L	4	5.25 ± 0.63	11	5.9 ± 1.8	NS
FSH peak, IU/L	4	11.6 ± 6.6	11	1.85 ± 0.07	NS
LH/FSH peak ratio	4	0.55 ± 0.35	11	3.2 ± 1.13	NS
Estradiol, pg/mL	9	2.5 ± 0.7	NA	NA	NA
Testicular volume index, cm^2^	NA	NA	33	7 ± 4.2	NA
Testosterone, ng/mL	NA	NA	28	0.46 ± 0.21	NA

*NA* not applicable, *NS* not significant

Karyotype was performed in 6 girls and was denoted 46 XX for all of them and in 4 boys and the only abnormality found was a 9p trisomy, usually associated with growth and puberty retardation [19].

Results were expressed as the means ± SD. The data are normally distributed and p values are corrected for multiple comparisons. Statistical analysis were performed using the Student test, except for comparing boys with bilineal to those with unilineal inheritance. Their data were not normally distributed because of the small sample; they were compared by the Mann-Whitney test.

## Results

Fourteen girls and thirty-four boys were included.

### Characteristics of the patients

The girls and boys had similar characteristics, excluding the ages at onset of puberty and at evaluation greater in boys, as expected (Table 1). The mean age of menarche among the girls’ mothers was significantly greater than among the boys’ mothers. Among the girls’ mothers, the menarche age was between 14 and 15 years in 2 and above 15 years in 10 (total 12, 86 %); among the boys’ mothers, the menarche age was between 14 and 15 years in 7 and above 15 years in 14 (total 21, 62 %).

Among the girls, 6 had a BMI ≤ -2 SDS and only one was considered obese with a BMI ≥ 2 SDS; among the boys, 6 had a BMI ≤ -2 SDS and 5 were considered obese. Among the girls, 8/14 had no breast development at the first evaluation. The uterus was prepubertal on the pelvic ultrasonography in the 5 evaluated.

### Mode of inheritance

**Fig. 1:**
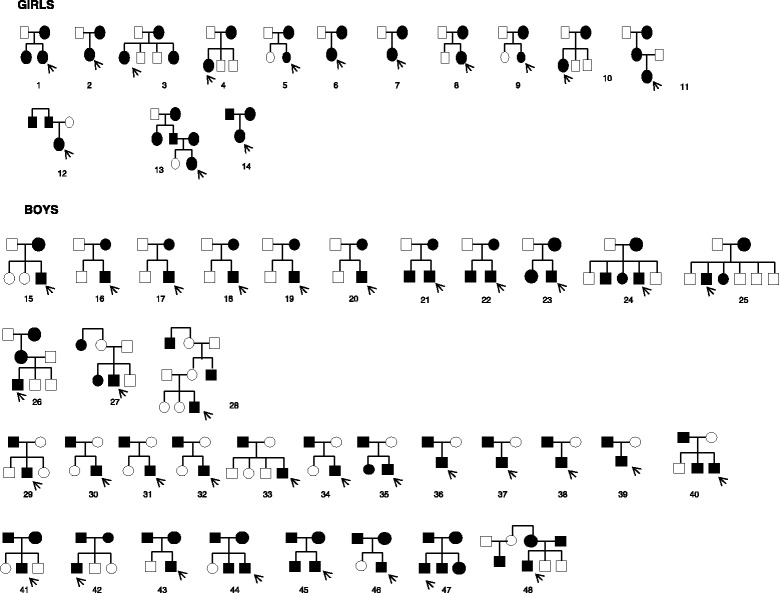
48 families with delayed puberty. Pedigrees exhibiting exclusively maternal inheritance are 1–11 (girls) and 15–28 (boys). Pedigrees exhibiting exclusively paternal inheritance are 12 (girls) and 29–40 (boys). Families showing both maternal and paternal inheritance are 13, 14 (girls) and 41–48 (boys). Solid squares represent affected male individuals and solid circles represent affected female individuals. The probands are indicated by an arrow in each case

**Fig. 2:**
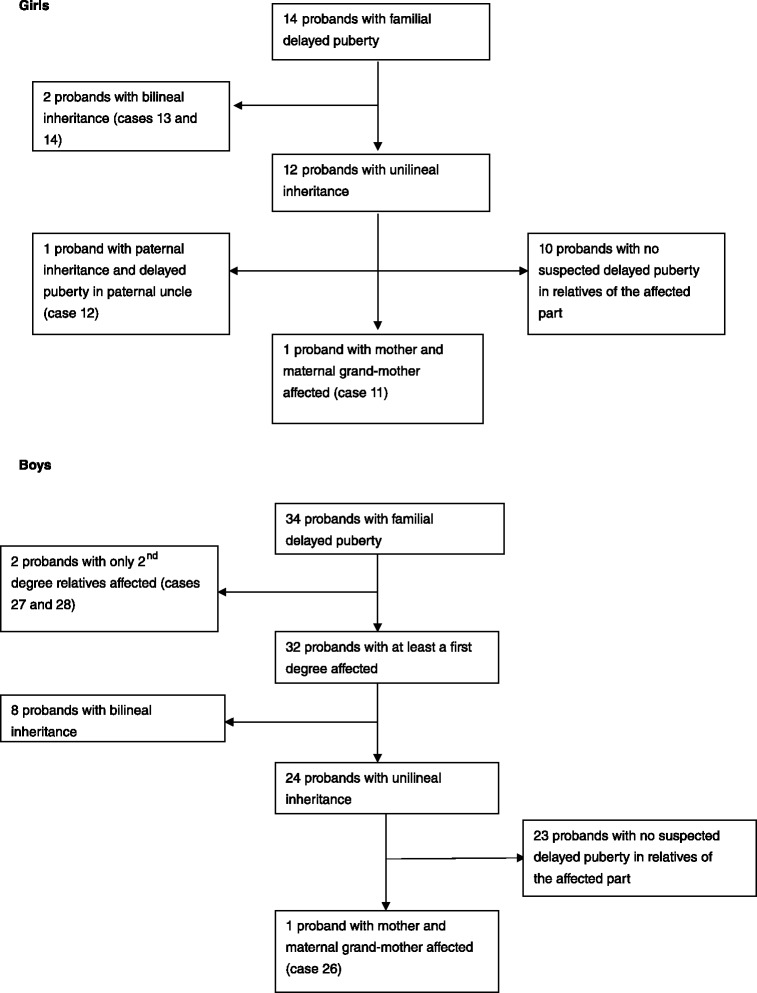
Familial clustering patterns of CDP in 14 girls probands and 34 boys probands

None of the families reported a history of consanguinity. Pedigrees of the 48 families are represented in Fig. 1 and familial clustering in Fig. 2. A total of 128 cases of CDP were identified, of whom 60 were affected girls and 68 affected boys. The male to female ratio for the probands was 2.4:1. It was 1:1.37 for the first degree relatives.

Of the 48 probands, 46 (96 %, 14 girls and 32 boys) had at least one affected 1^st^ degree relatives and 2 (4 %, cases 27 and 28) had only 2^nd^ degree relatives affected. There was no family in whom only siblings were affected.

In the girls, 11 families (79 %) exhibited exclusive maternal inheritance, 1 (7 %) paternal inheritance and 2 (14 %) presented both maternal and paternal inheritance. In the boys, 14 families (41 %) exhibited exclusive maternal inheritance, 12 (35 %) paternal inheritance and 8 (24 %) presented both maternal and paternal inheritance.

Of the 36 unilineal families, CDP was verified in three generations in only 3 of the patients (families 11, 26 and 28).

**Fig. 3:**
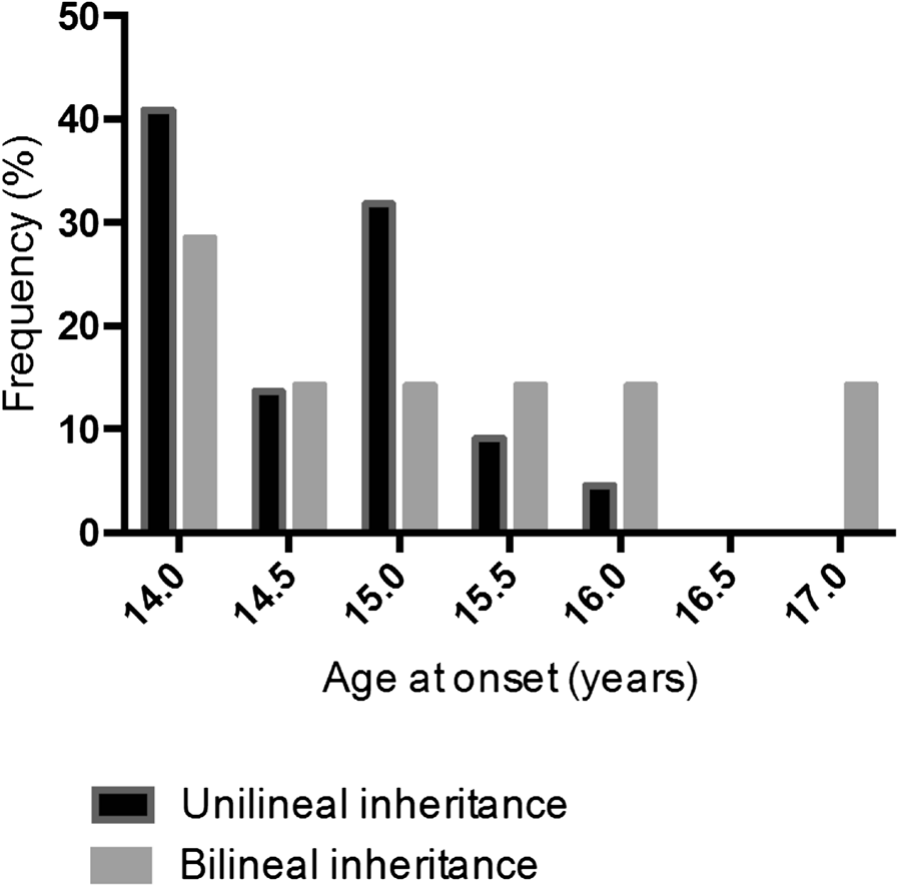
Boys with familial delayed puberty (*n* = 34): distribution of the ages at onset of puberty according to the unilineal or bilineal inheritance

In the boys with bilineal inheritance, the ages at onset of puberty (Fig. 3) and at evaluation were higher than in those with unilineal inheritance, but the difference was not significant (Table 2). They had similar plasma testosterone concentrations suggesting that these differences are not due to a difference in their pubertal development stages.

**Table 2:** Comparison of boys with familial constitutional delay of puberty according to the type (unilineal bilineal) of inheritance

	Unilineal inheritance (*n* = 26)		Bilineal inheritance (*n* = 8)		*p*
	*n*	m ± SD	*n*	m ± SD	
Age at onset, years	22	15.25 ± 0.35	7	16 ± 1.41	0.06
Age at evaluation, years	26	15.1 ± 0.42	8	16.05 ± 2.47	0.12
Testosterone, ng/mL	22	0.38 ± 0.35	6	0.32 ± 0.4	0.31

## Discussion

The results of this study suggest that despite various inheritance patterns among the pedigrees, there is evidence of autosomal dominant inheritance in CDP, with either complete or incomplete penetrance. In girls exclusive maternal inheritance seems to be the major mode of inheritance whereas for boys the mode of inheritance was almost equally maternal, paternal or bilineal. The results also suggest a more severe phenotype for boys with bilineal inheritance, but the difference did not reach statistical significance perhaps because of the small sample size.

Previous data, such as studies of twins, suggested that genetic factors account for 50–80 % of all variations in pubertal timing [4, 20]. Two extended studies addressed the segregation of CDP in families [7, 8] and showed an inheritance pattern compatible with autosomal dominant inheritance with complete or incomplete penetrance. Our study confirms this hypothesis and is the first to present all of the pedigrees included. We deliberately chose to include only familial CDP and no sporadic CDP, contrary to the previous studies, to focus on the mode of inheritance. Reproducing all of the pedigrees highlighted that 11/12 girls with CDP with unilineal inheritance had a mother who also had late puberty, while Wehkalampi et al. [7] reported a proportion of 5/12. We found no explanation for this discrepancy. The greater age at menstruation in the girls’ mothers than the boys’ mothers is probably linked to this quasi exclusive transmission by the mothers of the girls. Families with only siblings affected were rare: none in our series and 2 % in that reported by Wehkalampi et al. [7].

Our study is the first to suggest that there might be a more severe phenotype in boys with bilineal inheritance than in those with unilineal inheritance. The ages at onset of puberty and at evaluation were indeed higher in the boys with bilineal inheritance with a comparable testosterone level for boys with unilineal or bilineal inheritance, suggesting that this difference is not due to a difference in their pubertal development stages. This result has to be interpreted carefully regarding the small number of probands with bilineal inheritance analyzed.

In previous reports, the male to female ratio among CDP has ranged from 2:1 to 5:1 [5, 21, 22]. In our study, this ratio was 2.4:1 for the probands, but this male preponderance disappeared when calculated for the first degree relatives (1:1.37). In their study, Wehkalampi et al. [7] found this ratio at 1.2:1 . They suggested that the marked overrepresentation of boys with CDP at pediatric clinics may be biased. The authors noted that this potential biased overrepresentation could be linked to psychosocial reasons with boys being more interested in athletic performance and physical characteristics around puberty. Another explanation given by the authors is the trend toward earlier puberty in girls [23, 24], which has also been reported for boys [25].

Many studies of girls have demonstrated a link between more advanced breast development and higher BMI [26, 27] and that obese girls achieve menarche earlier than lean ones [28]. In boys, the association between pubertal development and adiposity has been less consistently reported with studies finding a positive association [25], lack of association [29] or negative association [30]. Children and adolescents with delayed puberty are typically relatively lean and this is true for the majority of our case series. However, it is possible that reporting the BMI SDS to BA, and not to chronological age, reduce the frequency of the lean patients as puberty itself results in increases in BMI. Some of the probands were overweight, especially the boys, which was already reported in CDP [5]. A recent genome-wide association study reported that BMI-increasing alleles correlated with earlier breast development in girls, whereas in boys some BMI-increasing alleles are associated with earlier and others with delayed sexual development [31]. This finding is consistent with epidemiological data and confirms that some mechanisms regulating pubertal onset in males and females might be different. These data are also consistent with our finding of different modes of inheritance in CDP for boys and girls; however, the high percentages of mothers that reached menarche after 14 years of age in both sexes suggest that if the low BMI has a role, it is additional to that of the familial factor. Alternatively, there could be a gene-environment interaction such that genetics could produce greater susceptibility to having pubertal timing affected by BMI.

What controls puberty remains largely unknown. The genome-wide association studies have shown that variants of more than 30 genes are associated with the age of menarche in humans [32]. Lomniczi et al. recently reported that the timing of female puberty was under the regulatory control of an epigenetic mechanism of transcriptional repression [33]. All of these reports confirm that the timing of puberty is controlled by many genes. This hypothesis is supported by the greater severity of the phenotype in boys with CDP with a bilineal family history, likely due to inheriting different puberty timing genes from both parents. This hypothesis should be confirmed in a larger cohort.

We acknowledge that our study has some limitations. We have no extended pedigrees. This may bias ascertainment/inclusion towards pedigrees with autosomal dominant inheritance. Relying on a parental report for the onset of puberty is not very accurate as many parents fail to notice when the puberty actually starts; however, even recalled information is valuable; studies suggest that even decades after the event, 75–90 % of women remember their age of menarche and 50 % of men remember the timing of their pubertal growth spurt within a year [34, 35]. Despite a 10-year inclusion period, the number of families included is much lower than for precocious and advanced puberty [36]. To try to confirm our data, further multicenter studies on larger cohorts should be conducted.

## Conclusions

This study confirms the autosomal dominant inheritance pattern in CDP in a monocentric pediatric cohort and the need for further genetic studies. Our data are also consistent with other studies suggesting that the mechanisms underlying the onset of puberty are different in girls and boys, and different modes of transmission in boys and girls were revealed. The greater severity of the phenotype in boys with a bilineal family history is likely due to inheriting different puberty timing genes from both parents. Future research should be directed at identifying such genes.

## Abbreviations

BA: bone age
BMI: body mass index
CDP: constitutional delay of puberty
FSH: follicle-stimulating hormone
GH: growth hormone
GnRH: gonadotropin-releasing hormone
IGF: insulin-like growth factor
LH: luteinising hormone
MRI: magnetic resonance imaging
SDS: standard deviation score
